# Leptospirosis in the Philippines: Confronting the Structural Roots of a Recurring Threat

**DOI:** 10.3390/pathogens14100963

**Published:** 2025-09-24

**Authors:** Jasmine Soco Interior, Kyrsten Jannae Jimenez Bigay-Iringan, Ria Nicole Dulaycan Bondad-Delson, Remigo Angelo Argayoso Iringan, Xiara Mei Sandoval Calderon, Anna Gabriele Perez Castro

**Affiliations:** 1 Mapua School of Medicine, Mapua University, Makati City 1002, Metro Manila, Philippines; 2 St. Luke’s Medical Center College of Medicine—William H. Quasha Memorial, Quezon City 1112, Metro Manila, Philippines; kjbigaymd@gmail.com (K.J.J.B.-I.); rnbdelson@gmail.com (R.N.D.B.-D.); angelo.iringan@gmail.com (R.A.A.I.); xiaramei.calderon@gmail.com (X.M.S.C.);

**Keywords:** leptospirosis, Philippines, infectious disease, flood-related infection, urban flooding, health inequities

## Abstract

Leptospirosis remains a pressing yet under-recognized public health burden in the Philippines with an alarming 43.45% rise in cases in early 2025. Outbreaks closely follow flooding, disproportionately affecting impoverished communities in informal, flood-prone settlements where poor sanitation, unsafe housing, and limited healthcare access compound vulnerability. Current responses remain largely hospital-based and reactive, straining resources during seasonal surges while leaving structural drivers unaddressed. This article calls for a shift to multisectoral, preventive strategies that reduce socioeconomic vulnerabilities through stronger intersectoral collaboration, investments in flood control and basic services, and enhanced digital surveillance. Without systemic reforms that integrate health, environment, and social policy, leptospirosis will continue to impose a recurring and inequitable burden on marginalized populations.

Leptospirosis, a resurgent but often neglected zoonotic disease, remains a significant public health burden in the Philippines. The Department of Health (DOH) recorded an alarming 43.4% rise in leptospirosis cases between January and July 2025 (3037 cases) compared to the same period in 2024 (2115 cases) [1,2]. Outbreaks consistently follow periods of intense rainfall and flooding, which enable rapid bacterial transmission.

Leptospirosis thrives in settings of poverty, which, in this context, refers to multidimensional deprivation including low household income, inadequate housing, unsafe water and poor sanitation, limited healthcare access, and insecure livelihoods. When combined with inadequate infrastructure and environmental degradation, these circumstances disproportionately affect vulnerable populations. Informal settlements in flood-prone areas of Metro Manila face the greatest risk due to chronic exposure to floodwater contaminated with rat urine, the primary reservoir of *Leptospira*. Poor drainage, inefficient waste management, and increasing climate-related disasters exacerbate these risks. Marginalized populations, such as those living in poverty, in informal settlements, or in areas with inadequate sanitation, face the greatest risk, with children, the elderly, and the malnourished experiencing higher exposure and worse outcomes, especially when co-infected with other endemic diseases like dengue or tuberculosis. Limited healthcare access, high treatment costs, and low health literacy delay diagnosis and treatment. Consequently, despite well-established risks, inconsistent public health responses continue to deepen community vulnerability.

Current control measures focus on free chemoprophylaxis, expanded treatment coverage through the Philippine Health Insurance Corporation (PhilHealth) [3], and public education. However, surges during the rainy season strain supplies and funding. Current responses remain predominantly hospital-based and reactive, with limited coordination between the health sector, disaster risk reduction agencies, flood control infrastructure, and community preparedness initiatives. This lack of integration undermines efforts to address systemic weaknesses in flood preparedness and community-level prevention, leaving a health system that responds to outbreaks as they occur but struggles to build long-term resilience against leptospirosis.

The Philippine experience reflects trends in other flood-prone nations, some of which have curbed outbreaks through proactive, multisectoral approaches. In Brazil, the 2024 Rio Grande do Sul floods prompted rapid deployment of field hospitals, mobile units, and digital surveillance tools [4]. Similarly, Fiji’s 2012 outbreak prompted integrated water, sanitation, and hygiene interventions [5]. These cases highlight the importance of community-driven strategies that address immediate health needs and broader social determinants, approaches that the Philippines could adapt to strengthen its response.

Leptospirosis will remain a recurring threat unless structural and socioeconomic factors are addressed. This demands intersectoral collaboration, long-term investments in sanitation and flood control infrastructure, and equitable access to clean water and housing. Public health efforts must also strengthen health education, ensure adequate supplies in health facilities, and enhance disease surveillance. In particular, the integration of digital epidemiological surveillance systems and structured, community-tailored health information campaigns could significantly improve outbreak preparedness and responses. At present, the Philippines lacks a unified, accessible national registry for leptospirosis and other climate-sensitive diseases, resulting in fragmented, delayed, and incomplete data reporting. Developing interoperable databases, linking hospitals, laboratories, and local health units, would enable real-time monitoring, faster outbreak detection, and evidence-based decision-making. Indeed, without systemic changes and sustained community engagement, existing initiatives will remain inadequate for mitigating the disease’s burden.
